# Dr. Henry Snowden

**Published:** 1894-02

**Authors:** 


AMERICAN JOURNAL

—OF—

DENTAL SCIENCE.

VOL. XXVII. BALTIMORE, FEBRUARY, 1894. NO. 10.

### Obituary.

### DR. HENRY SNOWDEN.

DR. HENRY SNOWDEN, one of the publishers of the AMERICAN JOURNAL OF DENTAL SCIENCE, and the senior member of the firm of Snowden & Cowman, died at his residence, 2104 Oak street, Baltimore, Maryland, January 16th, 1894, in the 74th year of his age.

Dr. Snowden was the son of Nicholas and Elizabeth Snowden, of Prince George County, Md., and was born September 29th, 1820, at Laurel, Md. His father owned large tracts of land and other property in that section of the State, and Dr. Henry Snowden was his fourth son. One brother, Dr. De Wilton Snowden, of Laurel, Md., and one sister, Eliza, (Sister Anna Maria,) of the Convent at Georgetown, D. C., still survive of the large family of Nicholas Snowden.

Dr. Henry Snowden was educated at Benjamin Hallowells' Boarding School, Alexandria, Virginia. Very early in life he exhibited a fondness for mechanics, and on the completion of his course of instruction at Alexandria, he returned to his home and served several years in the machine shops connected with the large cotton factory in Laurel, largely owned and controlled by his brother-in-law, Col. Horace Capron. When 26 years of age, and after receiving his interest from his father's estate, including several slaves, whom he set free, he came to Baltimore, and in connection with the old shipping and commission house of F. W. Brune and Sons, and others, formed a stock company at Canton, a suburb of Baltimore, which leased ground, and, under the supervision of Dr. Snowden, built a large cotton-duck factory together with many houses for the operatives.

He assumed the management of this industry, and a few years later built on an adjoining lot of ground another factory and dwellings, of which he was the sole owner, for the manufacture of cotton-laps. This factory was destroyed by fire shortly after it was completed, entailing great loss upon Dr. Snowden; and the following year, 1852, the Company's factory was also destroyed by fire, when Dr. Snowden turned his attention to dentistry, and became a student in the office of Dr. Joseph J. Stewart, who at that time was a young practitioner located at Canton. On leaving Dr. Stewart, Dr. Snowden opened an office in the city, where he practiced dentistry in all its details, becoming very proficient, especially in platina work.

About the year 1854 Dr. Snowden brought to Baltimore the first carded artificial teeth for use in his practice, and also for sale to other dentists.

At the suggestion of a number of his fellow practitioners of this city, Dr. Snowden, in 1856, gave up the practice of dentistry, and opened a depot for the sale of dental materials at No. 3 N. Liberty street, Baltimore. He conducted this business alone until May 1, 1860, when he associated with him as a partner his brother-in-law, Mr. Charles H. Cowman, and from that date the well known firm of "Snowden & Cowman" has existed. In 1866 this firm purchased the property No. 9 West Fayette street, where their dental depot is still located. Dr. Snowden was, therefore, closely identified with dentistry for many years, prior to his death, first as a practitioner and afterwards as an inventor of many useful appliances, and a manufacturer and dealer in dentists' supplies.

In 1859 Dr. Snowden invented various dental laboratory lathes, etc., which were great improvements over the old styles in use before that date. Among these were the "Amateur," "United States," "Hand and Foot," and "Table" lathes. In 1866 he invented the first complete iron frame dentist chair, with adjustable foot-board attached to the body of the chair, and the combined spittoon and instrument table, which were also attachments. Unfortunately for his own interests, as they were extensively adopted by other manufacturers, all of these, as well as a number of other useful inventions, were put upon the market without the protection of patents.

In 1874 Dr. Snowden turned his attention to hydraulics and other freight and passenger elevators, obtaining five different patents on such inventions.

In 1879 the firm of Snowden & Cowman erected a machine shop and foundry in South Baltimore for the construction of elevators and hoisting machinery, Dr. Snowden taking full charge of this branch of their business and to such work he devoted the latter years of his life. When about 18 years of age, Dr. Snowden joined the Methodist Episcopal Church at Laurel, and one year after his location in Baltimore (1847), he married Miss Mary E. Cowman, the daughter of John P. Cowman, Esq., of Alexandria, Va., who died in 1869, leaving no children. Shortly after the death of his wife, Dr. Snowden connected himself with St. Peter's P. E. Church, the rector of which was the Rev. Dr. J. E. Grammer, who officiated at his funeral, and in a most impressive and beautiful manner referred to his Christian life and character, and the good he had accomplished in behalf of the unfortunate and distressed. In 1878 Dr. Snowden married Miss M. Victoria Birkey of Baltimore, who still survives, with one son now 13 years old.

An attack of pneumonia three years before his death deprived Dr. Snowden of his usual vigor, and since that time his health continued to fail, although he continued to give attention to his business, and was active in the performance of his regular duties until his last illness, which confined him to bed about ten days, when he quietly and peacefully passed away to inherit the reward promised to those who have deserved well of their fellow-men.

Esteemed and respected in life and honored in death, Dr. Henry Snowden, like a sheaf that has ripened in the sunlight of many years, has been gathered into the Garner of God. Quiet and unobtrusive in manner, warm in his friendships, unfaltering in his devotion to every truth and trust, he was one who will be missed from every circle which he graced, and in every enterprise in which he was engaged. Dr. Snowden appreciated in all its strong import the divine command to love God and his neighbor, and in all the duties of domestic life he discharged the obligations involved in such relations with Christian fidelity. His uniform kindness of manners, his gentle heart, his warm affections and unobtrusive deportment secured the love and friendship of all who knew him.

He ever confronted error with mildness, and threw the mantle of charity over others' failings, believing that only as he forgave could he hope to be forgiven.

He was greatly devoted to the cause of humanity, and threw all his influence on the side of philantrophy, being actively engaged up to the time of his fatal illness in all measures conducive to the relief of the unfortunate.

Peace and honor to this good man's memory.
Let it grow green with years,
And blossom through the flight of ages.
Let the light stream on his deeds of love,
And in the book of fame the glorious
Record of his virtues write, and hold it up
To men and bid them claim a palm like his.

We take great pleasure in recording and perpetuating the following noble tribute from his revered and eloquent friend and pastor, Rev. Dr. Julius E. Grammer, who was for nearly twenty-eight years the esteemed Rector of St. Peter's P. E. Church, of Baltimore:

"Dr. Henry Snowden was identified with St. Peter's P. E. Church as a communicant, pew-holder and efficient worker in its various departments of philanthropic effort for many years. He was a man of ardent nature and decided convictions. He was first known to the Rector of that Church, for more than twenty-five years ago, and upon his first admission to the church, he came to the minister saying 'I want to work for God.' From that time he was a sedulous advocate of the claims of God upon his servants and people. He was conspicuous in unfurling and defending the banner of Temperance. He was an enthusiastic and intelligent advocate of Prohibition, and by his life and example did all in his power to advance that great and good cause.

The next enterprise dear to his heart was to help the wage-earner. He opened a house on Penna. avenue near Dolphin street, where operatives were asssisted in their handicraft by facilities in the use of machines and in the procuring of material for work. If Dr. Snowden had had means equal to his generous intentions in this regard, he would have been a man like Thornton in England, or like our own Cooper and Peabody in America. He was a philanthropist, a lover of his race, a friend of man and so like Abou Ben Adden, 'a friend of God.'

We find Dr. Snowden's name identified with a society having for its object the rescue and elevation of those who are fallen; and so strong was his faith and his love for God that he included all classes in the wide circle of his intercessions and labors for their amelioration. His tastes led him especially to select those fields of Christian effort where the need was greatest and where the struggle with poverty and sickness and sin and temptation in all its forms was the fiercest.

As a citizen of Baltimore he will be missed for the value of his moral influence, and personal integrity, and for the large contributions he made of his time and service and means according to his ability.

At his funeral a large company of representative citizens gathered, and among them were also to be seen the poor and dependent whom he had cheered and helped. His pastor spoke of him as a man of consistent and earnest Christian character, as a laborer in the vineyard of the Lord, and as a standard bearer, whose influence would long be felt in this community and in the church, where his example shone as a burning and a shining light.

The profession and department of practical mechanics with which he was identified has lost an inventive and active mind and a spirit of zealous enthusiasm consecrated to noble industry."

The following is contributed to this obituary notice by Mrs. E. B. Murdoch, the highly respected President of "The Fallen Women's Home," of Baltimore, Md., and beautifully portrays the philanthropy to which Dr. Snowden so generously and faithfully devoted his life :

"By the death of Henry Snowden, Esq., a great void is made in the ranks of workers for humanity in our city. Tender and sympathetic by nature, his heart-felt interest was especially directed to unfortunate, friendless and straying ones. So when twenty-five years ago a movement was inaugurated in this city for the reclamation of women who had wandered from the path of virtue and a Home was opened for such as would seek its shelter and help, Mr. Snowden accepted the invitation to become one of its Board of Directors. On the death of the 1st President, Richard Janney, he was chosen his successor, which position he retained till his death.

The Home was permanently located in 1874, Mr. Snowden generously aiding in the purchase of the building, No. 5 N. Exeter street. For the years following, till within the last two or three, when his health failed, he was regular in his attendance at the stated meetings, always manifesting the greatest interest in behalf of the inmates and freely giving to the support of the work.

It seemed, indeed, to be an *ever* present interest with him, for, we are told, that at the family altar, he never failed to ask for the blessing of God to rest upon these friendless, unfortunate ones, and upon those who were seeking to rescue and to save them. His last visit to the "Home" was on the afternoon of Dec. 31st, the last Sabbath and the last day of the year. He spoke very tenderly of the season and the uncertainty of life, urging his hearers to live just such a life as should prepare them for a happy death!

Yes, he will be greatly missed, but he has entered into his rest, while the gracious influence of his life remains with us as a blessed heritage and memory!"—MRS. E. B. M."

F. J. S. G.

